# Lateral hypothalamic fast-spiking parvalbumin neurons modulate nociception through connections in the periaqueductal gray area

**DOI:** 10.1038/s41598-019-48537-y

**Published:** 2019-08-19

**Authors:** Justin N. Siemian, Cara B. Borja, Sarah Sarsfield, Alexandre Kisner, Yeka Aponte

**Affiliations:** 1 Neuronal Circuits and Behavior Unit, National Institute on Drug Abuse Intramural Research Program, National Institutes of Health, Baltimore, MD 21224-6823 USA; 2 The Solomon H. Snyder Department of Neuroscience, Johns Hopkins University School of Medicine, Baltimore, MD 21205 USA

**Keywords:** Synaptic transmission, Neural circuits

## Abstract

A pivotal role of the lateral hypothalamus (LH) in regulating appetitive and reward-related behaviors has been evident for decades. However, the contributions of LH circuits to other survival behaviors have been less explored. Here we examine how lateral hypothalamic neurons that express the calcium-binding protein parvalbumin (PVALB; LH^PV^ neurons), a small cluster of neurons within the LH glutamatergic circuitry, modulate nociception in mice. We find that photostimulation of LH^PV^ neurons suppresses nociception to an acute, noxious thermal stimulus, whereas photoinhibition potentiates thermal nociception. Moreover, we demonstrate that LH^PV^ axons form functional excitatory synapses on neurons in the ventrolateral periaqueductal gray (vlPAG), and photostimulation of these axons mediates antinociception to both thermal and chemical visceral noxious stimuli. Interestingly, this antinociceptive effect appears to occur independently of opioidergic mechanisms, as antagonism of μ-opioid receptors with systemically-administered naltrexone does not abolish the antinociception evoked by activation of this LH^PV^→vlPAG pathway. This study directly implicates LH^PV^ neurons in modulating nociception, thus expanding the repertoire of survival behaviors regulated by LH circuits.

## Introduction

The diverse collection of genetically-distinct cell types in the lateral hypothalamus (LH) is crucial for orchestrating a variety of motivated behaviors that facilitate survival^1,2^. Over the past decade, tremendous progress has been made on new methods that allow systematic characterization of the function and connectivity of these heterogeneous neuronal subtypes. Recent studies using optogenetics and chemogenetics showed that activation of LH GABAergic neurons promotes feeding and reward-seeking behaviors^3^, whereas activation of LH glutamatergic neurons suppresses feeding and is aversive^4^. Moreover, LH GABAergic and glutamatergic neurons project to the ventral tegmental area (VTA), and activation of these pathways positively and negatively influences feeding behavior, respectively^5^, as well as other motivated behaviors such as social interaction^6^. While studies such as these have begun to identify LH circuits that regulate food intake and reward-related behaviors, less attention has been given to the contributions of genetically-identified LH circuits in modulating nociceptive behaviors.

Pioneering experiments using electrical or chemical stimulation and traditional electrophysiology methods showed that activation of the LH suppresses nociception^7–9^ and triggers an increase of neuronal activity in the periaqueductal gray area (PAG)^10^. Furthermore, LH neurons respond to both noxious stimuli and local microinjection of morphine^11,12^. Thus, these studies suggest that the LH and LH−PAG pathways can modulate nociceptive behaviors. While pharmacological experiments have demonstrated that tachykinin 1 (TACR1; *i.e*. neurokinin 1 receptor), hypocretin 2 (HCRT2), and cannabinoid 1 (CNR1) receptors in the PAG may be important for LH stimulation-induced antinociception^13,14^, the specific, genetically-identified cell types within the LH encoding nociception have yet to be determined.

Recently, we demonstrated that a small cluster of neurons in the LH identified by the expression of the calcium-binding protein parvalbumin (PVALB; LH^PV^) have a fast-spiking action potential phenotype, release the excitatory neurotransmitter glutamate, and provide excitatory control of local neuronal circuits within the LH^15^. Moreover, our findings and those of others showing that LH^PV^ neurons send long-range projections to several brain regions involved in reward, motivation, and nociception^15,16^ suggest that these neurons may be essential for regulating survival behaviors. In further support, chemical ablation of LH^PV^ neurons in rats^17^ or conditional inactivation of glutamatergic signaling of parvalbumin-expressing neurons in mice^18^ decreased vocalization and locomotion, respectively. In addition, decreased thermal nociception and increased social dominance were also observed in these knockout mice^18^. However, a potential confounding factor in these studies might be the selective deletion of vesicular glutamate transporter 2 (*Slc17a6, i.e. Vglut2*) targeted to all neurons that co-express PV (PV^+^/VGLUT2^+^) throughout the entire brain. Therefore, other brain circuits that contain PV^+^/VGLUT2^+^ neurons^19^ may account for the behavioral responses observed. Thus, studies have not yet demonstrated the specific contributions of LH^PV^ neurons to behavior.

Because anterograde and retrograde tracing studies have demonstrated that LH^PV^ neurons densely project to the vlPAG, mainly to the supraoculomotor (Su3) region^15,16^, and neuronal circuits in the vlPAG regulate nociception^20,21^ as well as fear- and defensive-related behaviors^22–25^, we sought to directly determine whether LH^PV^ neurons modulate these types of survival behaviors. We first used optogenetics to selectively manipulate LH^PV^ neuronal activity and determine effects on hot plate, open field, and elevated plus maze behavior. We then combined optogenetics and electrophysiology to demonstrate that LH^PV^ neurons are synaptically connected to cells within the vlPAG and that activation of this LH^PV^→vlPAG pathway also modulates nociceptive responses to both thermal and chemical visceral noxious stimuli. Finally, since the PAG is an important site for opioid-mediated analgesia^20,21,26,27^, we investigated whether modulation of nociception by the LH^PV^→vlPAG pathway involved opioidergic mechanisms.

## Results

### LH^PV^ neuronal activity modulates nociceptive behaviors

**Figure 1:**
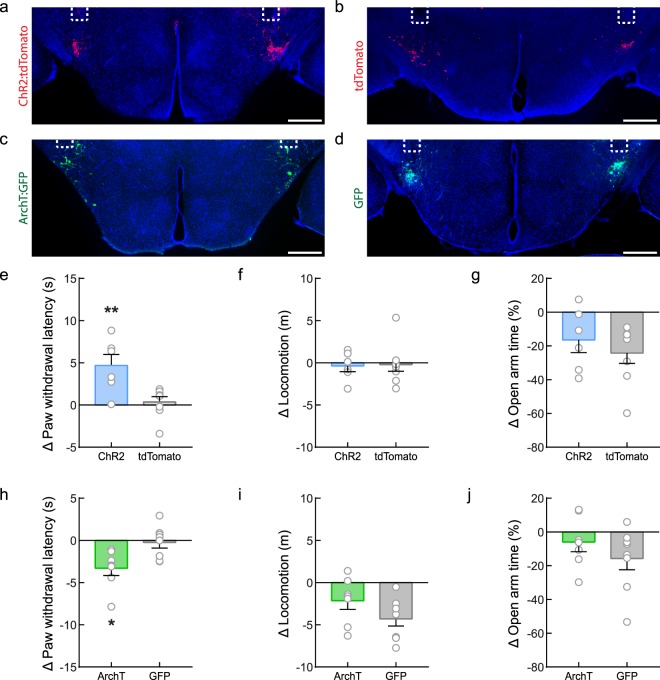
Bidirectional modulation of nociceptive behaviors by LH^PV^ neurons. (**a**,**b**) Representative images showing bilateral expression of (**a**) ChR2:tdTomato (red) and (**b**) tdTomato (control; red) in LH^PV^ neurons. (**c**,**d**) Representative images depicting (**c**) ArchT:GFP (green) and (**d**) GFP (control; green) expression. Optical fibers were implanted above the LH bilaterally (white dotted lines). Scale bars: 500 μm. See also Supplementary Fig. S1. (**e**) Change in paw withdrawal latency in response to a 51 °C hot plate (PWL_HP_) during photostimulation (450 nm, 10–15 mW, 50 Hz, 5-ms pulses) from baseline (∆ PWL_HP_ = PWL_HP_ during photostimulation – PWL_HP_ before photostimulation). Photostimulation of LH^PV^:ChR2 mice (blue) evoked a significantly greater increase in PWL_HP_ compared to control LH^PV^:tdTomato (gray) mice; **p = 0.007. (**f**) Photostimulation did not affect open field locomotor activity (∆ locomotion = distance traveled during photostimulation – distance traveled before photostimulation) between groups; *p* = 0.84. (**g**) Photostimulation did not alter the time spent in the open arms of an elevated plus maze (∆ open arm time = percentage time spent in open arms during photostimulation – percentage time spent in open arms before photostimulation) in both LH^PV^:ChR2 and control mice; *p* = 0.44. (**h**) Photoinhibition (520 nm, 10–15 mW, constant 3 min) of LH^PV^:ArchT mice (green) evoked a significantly greater decrease in PWL_HP_ compared to control LH^PV^:GFP (gray) mice; *p = 0.0145. (**i**) Photoinhibition did not alter locomotor activity in LH^PV^:ArchT compared to control mice; *p* = 0.13. (**j**) Photoinhibition did not alter the time spent in the open arms of an elevated plus maze in both LH^PV^:ArchT and control mice; *p* = 0.30. Bars represent mean ± s.e.m.; circles indicate data from individual mice.

We first investigated whether LH^PV^ neurons regulate nociceptive behaviors by measuring the latency to paw withdrawal during a hot plate assay (PWL_HP_) while acutely activating or inhibiting these neurons using optogenetics. To specifically target and manipulate LH^PV^ neurons, we bilaterally injected a Cre recombinase-dependent viral vector driving the expression of either channelrhodopsin (ChR2:tdTomato; light-sensitive neuronal activator) or archaerhodopsin (ArchT:GFP; light-sensitive neuronal silencer) into the LH of *Pvalb*^*Cre*^ transgenic mice^28^. Next, we implanted optical fibers bilaterally above these neurons (Fig. 1a–d and Supplementary Fig. S1). Following recovery, mice were tested for thermal nociception in two consecutive 3-min epochs before and during photostimulation (5-ms light pulses, 50 Hz). We found that optogenetic activation of LH^PV^ neurons evoked a significantly greater increase in PWL_HP_ from baseline compared to LH^PV^:tdTomato control mice (Fig. 1e; *n* = 6 LH^PV^:ChR2, *n* = 8 LH^PV^:tdTomato; unpaired Student’s *t* test, *t*(12) = 3.25, **p = 0.007). Importantly, open field locomotion was not affected by photostimulation of these neurons (Fig. 1f; *n* = 6 LH^PV^:ChR2, *n* = 8 LH^PV^:tdTomato; unpaired Student’s *t* test, *t*(12) = 0.21, *p* = 0.84), suggesting that the increases in PWL_HP_ were not due to motor impairment. Because LH^PV^ neurons densely project to the vlPAG^15,16^ and previous studies have shown that neuronal circuits in the vlPAG regulate nociception, anxiety, and fear-related defensive behaviors^22–25^, we also sought to determine whether LH^PV^ neurons modulate the time spent in the open arms of an elevated plus maze, a common assay for anxiety-like behavior. We found that photostimulation of LH^PV^ neurons did not significantly affect the time spent in the open arms of the maze (Fig. 1g; *n* = 6 LH^PV^:ChR2, *n* = 8 LH^PV^:tdTomato; unpaired Student’s *t* test, *t*(12) = 0.80, *p* = 0.44) or open or closed arm entries (Supplementary Fig. S2), suggesting that these neurons may not regulate anxiety-related behaviors.

Next, we found that photoinhibition of LH^PV^ neurons evoked a significantly greater decrease in PWL_HP_ from baseline compared to LH^PV^:GFP control mice (Fig. 1h; unpaired Student’s *t* test, *n* = 7 LH^PV^:ArchT, *n* = 8 LH^PV^:GFP; *t*(13) = 2.82, *p = 0.0145). Moreover, no differences in locomotor activity (Fig. 1i; unpaired Student’s *t* test, *n* = 7 LH^PV^:ArchT, *n* = 8 LH^PV^:GFP; *t*(13) = 1.61, *p* = 0.13), time spent in the open arms of an elevated plus maze (Fig. 1j; unpaired Student’s *t* test, *t*(13) = 1.09, *p* = 0.30), or open or closed arm entries (Supplementary Fig. S2) were observed during photoinhibition of LH^PV^ neurons as compared to control mice. Together, these results demonstrate a bidirectional modulation of thermal nociception by LH^PV^ neurons.

### LH^PV^ neurons regulate nociceptive processing via projections to the vlPAG

**Figure 2:**
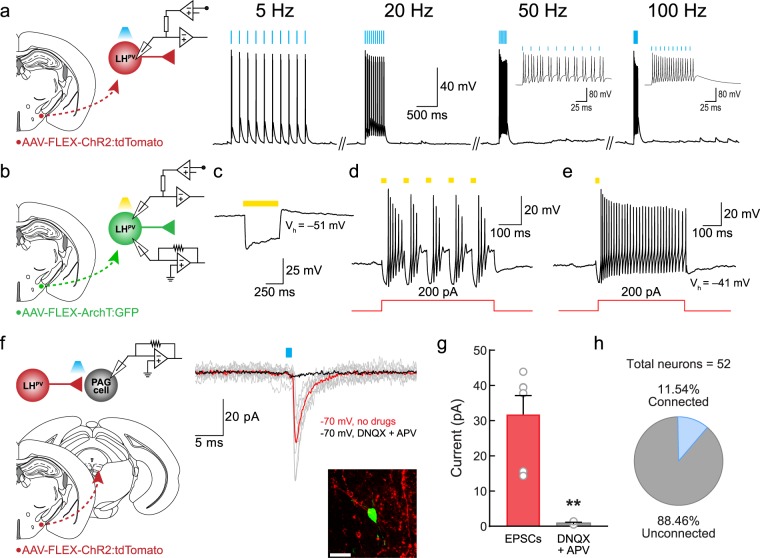
LH^PV^ neurons provide excitatory input to neurons in the vlPAG. (**a**) (*Left Panel*) Schematic of whole-cell current-clamp recordings of ChR2-expressing LH^PV^ neurons. (*Right Panels*) Representative traces from a ChR2-expressing LH^PV^ neuron responding to photostimulation (470 nm light) at 5 Hz (100 ± 0% fidelity), 20 Hz (103 ± 9% fidelity), 50 Hz (83 ± 9% fidelity), and 100 Hz (75 ± 15% fidelity). (*Insets*) Expanded 50 Hz and 100 Hz traces. (**b**) Schematic of whole-cell current-clamp and voltage-clamp recordings of ArchT-expressing LH^PV^ neurons. (**c**) Representative traces from an ArchT-expressing LH^PV^ neuron responding to photoinhibition by a 500-ms yellow (590 nm) light pulse during a depolarizing current injection of 100 pA, (**d**) consecutive 20-ms light pulses delivered at 20 Hz during continuous depolarizing current injection of 200 pA, and (**e**) a single 20-ms light pulse delivered at the beginning of current injection. Note that a 500-ms light pulse evoked an inward hyperpolarizing current and both single and consecutive 20-ms light pulses blocked firing activity of LH^PV^-ArchT^+^ neurons triggered by a depolarizing current injection. (**f**) (*Left Panel*) Schematic of ChR2-assisted circuit mapping for LH^PV^-ChR2^+^ axonal projections in the vlPAG. (*Middle Panel*) Representative traces of excitatory postsynaptic currents (EPSCs; gray) evoked by photostimulation (5-ms light pulses) of LH^PV^-ChR2^+^ axons in the vlPAG before (red trace) and after (black trace) bath application of DNQX and APV (AMPA-R and NMDA-R antagonists). Traces are the average of ten consecutive sweeps. (*Right Panel*) Recorded vlPAG neuron filled with biocytin (green) surrounded by ChR2:tdTomato-expressing LH^PV^ axons (red). Scale bar: 20 μm. Schematic images adapted from Franklin and Paxinos^38^. (**g**) Summary bar graph showing the amplitude of excitatory postsynaptic currents (EPSCs) evoked by photostimulation of LH^PV^-ChR2^+^ axons in the vlPAG before (EPSCs; red) and after (gray) bath application of 10 µM DNQX and 50 µM APV (AMPA-R and NMDA-R antagonists). Bars represent mean ± s.e.m.; circles indicate data from individual vlPAG neurons. (**h**) Summary chart showing the percentage of vlPAG neurons that exhibited a synaptic response during photostimulation of LH^PV^-ChR2^+^ axons.

LH^PV^ neurons send long-range projections to the vlPAG, mainly to the supraoculomotor (Su3) nucleus in the PAG^15,16^, where they may regulate nociceptive processing by modulating the activity of these downstream neuronal circuits. However, functional synapses between LH^PV^ and vlPAG neurons have not been examined. To determine whether LH^PV^ neurons are synaptically connected to cells within the vlPAG, we performed ChR2-assisted circuit mapping in brain slices (CRACM)^29^. As described above, we specifically targeted ChR2 to LH^PV^ neurons with a Cre recombinase-dependent viral vector. We first performed whole-cell current-clamp recordings to demonstrate that LH^PV^ neurons respond to photostimulation with high fidelity even at high frequencies of up to 100 Hz (Fig. 2a, *n* = 4 neurons). Additionally, we performed both whole-cell current-clamp and voltage-clamp recordings to verify that ArchT-expressing LH^PV^ neurons are silenced during photoinhibition (Fig. 2b–e, *n* = 3 neurons). Next, we performed whole-cell recordings from individual neurons (*n* = 52 neurons) in the Su3 vlPAG under voltage-clamp configuration and observed that photostimulation of ChR2-expressing LH^PV^ axons evoked excitatory postsynaptic currents (Fig. 2f,g; EPSCs, 31.8 ± 5.4 pA; latency, 2.5 ± 0.3 ms) in vlPAG neurons (*n* = 6 neurons from *n* = 5 mice; 11.5% connected, Fig. 2h). These EPSCs were significantly attenuated by selective antagonists of glutamate receptors (Fig. 2f,g, EPSCs, 0.9 ± 0.2 pA; unpaired Student’s *t* test, *t*(7) = 3.92, **p = 0.006), confirming that LH^PV^ neurons provide functional excitatory synaptic inputs to the vlPAG.

**Figure 3:**
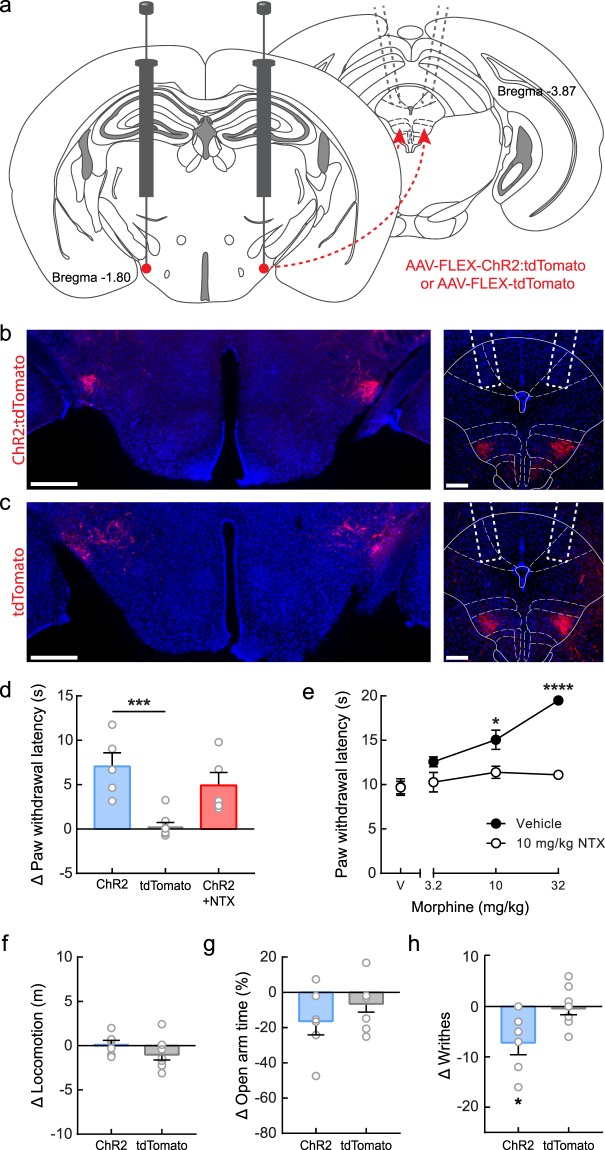
Activation of the LH^PV^→vlPAG circuitry promotes antinociception. (**a**) Schematic representation of ChR2 viral injections in the LH and optical fiber placement in the vlPAG (gray dotted lines). Schematic images modified from Franklin and Paxinos^38^. (**b**,**c**) Representative images showing bilateral expression of (**b**) ChR2:tdTomato (red) and (**c**) tdTomato (control; red) in LH^PV^ neurons. Scale bars: 500 μm. Optical fibers were implanted above the vlPAG bilaterally (white dotted lines represent average placement). Scale bars: 200 μm. See also Supplementary Fig. S3. (**d**) Photostimulation evoked a significantly greater increase in PWL_HP_ in LH^PV^:ChR2→vlPAG mice (blue) compared to control LH^PV^:tdTomato→vlPAG mice (gray); Bonferroni multiple comparisons test, ***p = 0.0007. This increase was not significantly affected by pre-treatment with naltrexone (NTX; 10 mg/kg s.c., μ-opioid receptor antagonist), *p* = 0.43. (**e**) NTX (10 mg/kg s.c.) significantly attenuated morphine-induced antinociception when tested in control mice. Bonferroni post-test, *p = 0.0167, ****p < 0.0001. (**f**) Photostimulation did not evoke changes in locomotor activity in LH^PV^:ChR2→vlPAG or control mice during the open field locomotion test; *p* = 0.20. (**g**) Photostimulation of LH^PV^:ChR2→vlPAG mice and control mice did not affect the time spent in the open arms of an elevated plus maze; *p* = 0.27. (**h**) Writhing responses to an i.p. injection of 0.6% acetic acid were measured over a 30-min period with alternating 3-min epochs paired with photostimulation (450 nm, 10–15 mW, 50 Hz, 5-ms pulses). LH^PV^:ChR2→vlPAG mice writhed significantly less during photostimulation than control LH^PV^:tdTomato→vlPAG mice; *p = 0.0212. Bars represent mean ± s.e.m.; circles indicate data from individual mice.

We next examined whether this LH^PV^→vlPAG pathway promotes antinociception by photostimulating ChR2-expressing LH^PV^ axon terminals within the vlPAG during the hot plate test. To test this, LH^PV^ neurons were targeted with ChR2 using a Cre recombinase-dependent viral vector and optical fibers were implanted bilaterally above the vlPAG to specifically activate the LH^PV^ axonal projections (Fig. 3a–c and Supplementary Fig. S3). We found that optogenetic activation of the LH^PV^→vlPAG pathway evoked a significantly greater increase in PWL_HP_ compared to LH^PV^:tdTomato→vlPAG control mice (Fig. 3d; *n* = 5 LH^PV^:ChR2→vlPAG mice, *n* = 8 LH^PV^:tdTomato→vlPAG mice; one-way ANOVA Bonferroni multiple comparisons test, ***p = 0.0007) suggesting that LH^PV^ neurons regulate nociceptive processing through connections in the vlPAG. Since neuronal circuits in the PAG are crucial for opioid-induced analgesia^20,21,26,27^, we sought to examine whether the LH^PV^→vlPAG pathway engages opioidergic mechanisms to promote antinociception. To test this, LH^PV^:ChR2→vlPAG mice were injected subcutaneously (s.c.) with the μ-opioid receptor antagonist naltrexone (10 mg/kg, s.c.) 30 min prior to photostimulation during the hot plate test. We found that naltrexone pre-treatment did not affect the photostimulation-induced increase in PWL_HP_ (Fig. 3d; *n* = 5 mice, one-way ANOVA Bonferroni multiple comparisons test, *p* = 0.43), suggesting that the antinociception encoded by the LH^PV^→vlPAG circuit likely does not involve an opioidergic mechanism. Importantly, the same dose of naltrexone significantly attenuated morphine-induced antinociception during the hot plate test, demonstrating sufficient opioid receptor antagonism (Fig. 3e; *n* = 8 mice, two-way repeated-measures ANOVA morphine × naltrexone interaction, *F*_3,21_ = 9.57, *p* = 0.0003; Bonferroni post-test, *p = 0.0167, ****p < 0.0001). Moreover, no changes in locomotor activity (Fig. 3f; *n* = 6 LH^PV^:ChR2→vlPAG mice, *n* = 8 LH^PV^:tdTomato→vlPAG mice; unpaired Student’s *t* test, *t*(12) = 1.36, *p* = 0.20), the time spent in the open arms of an elevated plus maze (Fig. 3g; *n* = 6 LH^PV^:ChR2→vlPAG mice, *n* = 8 LH^PV^:tdTomato→vlPAG; unpaired Student’s *t* test, *t*(12) = 1.15, *p* = 0.27), or open or closed arm entries (Supplementary Fig. S2) were observed during LH^PV^→vlPAG photostimulation.

Finally, we investigated whether this LH^PV^→vlPAG pathway was specific to thermal nociception. For this, we tested the effects of LH^PV^→vlPAG photostimulation on acetic acid-induced writhing, a model of chemical visceral nociception^30–32^. We observed a significant decrease in writhing during photostimulation of the LH^PV^:ChR2→vlPAG mice compared to the tdTomato control mice (Fig. 3h; *n* = 6 LH^PV^:ChR2→vlPAG mice, *n* = 8 LH^PV^:tdTomato→vlPAG mice; unpaired Student’s *t* test, *t*(12) = 2.65, *p = 0.0212). Together, these findings identify LH^PV^ neurons as a novel functional component of the LH glutamatergic circuitry modulating thermal and chemical nociception through connections with neuronal circuits within the vlPAG in mice.

## Discussion

The LH is a critical hub for regulating appetitive and reward-related behaviors^1^. Due to rapid technical advances for cell-type-specific manipulations linking neuronal circuits to behavioral functions, the contributions of lateral hypothalamic circuits to other survival behaviors, such as nociception, that have received more limited attention are starting to be revealed. Pivotal work in rodents revealed that gross manipulations of the LH modulate nociceptive behaviors^10,11^. Moreover, pharmacological approaches have shown that receptors in the PAG such as neurokinin 1 receptor (TACR1), hypocretin receptor 2 (HCRTR2), and cannabinoid receptor 1 (CNR1)^13,14^ as well as α_2_-adrenoceptors in the spinal cord^33^, are important for LH stimulation-induced antinociception. However, the specific genetically-identified cell types within the LH and their anatomical connections encoding nociception have yet to be determined. Here, we demonstrate for the first time that LH^PV^ neurons, representing a novel functional component of the LH glutamatergic circuitry, modulate nociception in mice through connections with the vlPAG.

Recently, we showed that LH^PV^ neurons have a fast-spiking phenotype like the inhibitory GABAergic PV neurons that are widely found in the neocortex and hippocampus^15^. However, unlike these GABAergic fast-spiking PV neurons, LH^PV^ neurons release the excitatory neurotransmitter glutamate and provide excitatory control of local LH neuronal circuits^15^. Moreover, our work and that of others^15,16^ showed that LH^PV^ neurons also send long-range projections to several brain regions involved in nociception, reward, and motivation, including the vlPAG and the lateral habenula (LHb). However, despite this detailed LH^PV^ axonal projection map, studies had not yet demonstrated the specific contributions of long-range LH^PV^ axonal projections to behavior.

Here, we find that increased LH^PV^ neuronal activity suppresses nociception to a noxious thermal stimulus in mice, whereas inhibition of neuronal activity causes hypersensitivity. Interestingly, previous studies showed that mice with conditional deletion of glutamatergic signaling in PV-expressing neurons display a phenotype characterized by decreased thermal nociception, deficits in locomotion, and increased social dominance^18^. However, as stated in the study, the observed findings are suggestive as genetic compensation in response to a gene knockout is a widespread phenomenon^34^. Furthermore, the selective deletion of *Vglut2* occurred in all neurons that express PV throughout the brain, and thus, other brain circuits that contain PV^+^/VGLUT2^+^ neurons^19^ could potentially contribute to the behavioral outcomes. In contrast, our finding that cell type-specific LH^PV^ neuronal activation or inhibition attenuates or promotes thermal nociception, respectively, directly implicates LH^PV^ neurons in the regulation of nociceptive processing. Moreover, since accurate measurements of paw withdrawal in response to a hot plate depend on functional locomotor activity and open field locomotion was not affected by manipulating the activity of LH^PV^ neurons, our data suggest that generalized locomotor changes did not account for the facilitated or inhibited nociceptive responses. Furthermore, anxiety-like behavior as measured by the elevated plus maze does not seem to be affected by optogenetically manipulating the activity of LH^PV^ neurons. Our findings are consistent with a previous study showing that the entries and time spent in the open sectors of an elevated zero maze were not significantly affected in mice with conditional inactivation of glutamatergic signaling in PV-expressing neurons in comparison to their wild-type littermates^18^. In addition, that study reported no changes in the number of fecal boli deposited during the elevated zero maze assay or in Morris water maze thigmotaxis. While the complex question of stress-related effects on nociception should be further investigated in future work, the current data suggest that the role of LH^PV^ neurons is likely specific to nociception, in contrast to this previous study^18^.

While our previous work and that of others demonstrated that LH^PV^ neurons send axonal projections to the vlPAG^15,16^, studies had not yet investigated whether there were functional synapses. At the circuit level, our work demonstrates that LH^PV^ neurons provide excitatory input to circuits within the vlPAG using channelrhodopsin-assisted circuit mapping (CRACM). These results also extend findings from previous studies showing the glutamatergic nature of LH^PV^ neurons^15,35^. Importantly, our findings that photostimulation of ChR2-expressing LH^PV^ neurons can trigger action potentials at frequencies of up to at least 100 Hz with high fidelity support our previous characterization of these neurons as fast-spiking^15^. Moreover, our findings that activation of this LH^PV^→vlPAG pathway attenuates both thermal and visceral nociception suggest that this pathway not only modulates responses to acute reflex-withdrawal assays of pain but also to an ongoing noxious stimulus. Similar to our results for somatic LH^PV^ photostimulation, LH^PV^→vlPAG activation did not alter locomotor or anxiety-related behavior, supporting the idea that this pathway may be specific to nociception.

Furthermore, the antinociception encoded by the LH^PV^→vlPAG circuitry likely occurs through non-opioidergic mechanisms as we find that antagonism of μ-opioid receptors by naltrexone administration does not affect the behavioral response to LH^PV^ neuronal activation. Together, these findings identify the LH^PV^→vlPAG pathway as an attractive translational target for pain therapies. However, further analyses are required to determine the specific neuronal types within the vlPAG that form synaptic connections with LH^PV^ neurons to regulate nociceptive processing. Interestingly, a recent study in mice using selective chemogenetic manipulation of neuronal activity in the vlPAG demonstrated that activation of glutamatergic or GABAergic neurons suppresses or potentiates nociception, respectively^21^. Thus, it is possible that LH^PV^ neurons modulate nociceptive processing by excitatory control of glutamatergic neurons in the vlPAG to attenuate nociception. Alternatively, LH^PV^ neurons may function by activating inhibitory interneurons in the vlPAG that provide local inhibitory control of vlPAG GABAergic neurons to suppress nociception. Future studies will examine the nature of LH^PV^ connectivity within the vlPAG and explore other LH^PV^ projection fields that may contribute to antinociception.

In summary, our study implicates LH^PV^ neurons in the regulation of nociception through interaction with downstream circuits in the vlPAG, further characterizing and expanding the list of survival behaviors regulated by lateral hypothalamic circuits.

## Materials and Methods

### Animals

All experimental protocols were conducted in accordance with the National Institutes of Health Guide for the Care and Use of Laboratory Animals and with the approval of the National Institute on Drug Abuse Animal Care and Use Committee. Male and female heterozygous *Pvalb*^*Cre*^ mice (RRID:IMSR_JAX:008069; C57BL/6J background, Strain 8069, The Jackson Laboratory, Bar Harbor, ME, USA) were used in this study. Mice were maintained at the National Institute on Drug Abuse animal facility under standard housing conditions. Up to five mice of the same sex were group housed under a 12-hour light-dark cycle at 20–24 °C and 40–60% humidity with free access to water and food (PicoLab Rodent Diet 20, 5053 tablet, LabDiet/Land O’Lakes Inc., St. Louis, MO, USA). For behavior experiments, six- to eight-week-old male and female mice (∼18–25 g) were randomly assigned to experimental groups while maintaining littermate or age-matched and gender-matched controls. Following stereotaxic surgeries, mice were individually housed.

### Stereotaxic viral injection

For behavioral experiments using optogenetics, mice were anesthetized with isoflurane and placed onto a stereotaxic apparatus (David Kopf Instruments, Tujunga, CA, USA). After exposing the skull by a minor incision, small holes (<1 mm diameter) were drilled bilaterally for virus injection. For experiments targeting parvalbumin neurons in the lateral hypothalamus (LH^PV^), 25 nl of an adeno-associated virus (rAAV2/1-CAG-FLEX-*rev*-ChR2-tdTomato, titer: 6.9 × 10^12^ GC/ml, RRID:Addgene_18917; rAAV2/1-CAG-FLEX-tdTomato, titer: 4.5 × 10^12^ GC/ml, RRID:Addgene_51503^36^; rAAV2/9-CAG-FLEX-ArchT-GFP, titer: 4.7 × 10^12^ GC/ml, RRID:Addgene_28307^37^; or rAAV2/9-CAG-FLEX-GFP, titer: 3.3 × 10^12^ GC/ml, RRID:Addgene_51502^36^) was injected bilaterally (rate: 25 nl/min) into the LH of *Pvalb*^*Cre*^ mice (bregma, −1.80 mm; midline, ±1.40 mm; skull surface, −5.40 mm) by a pulled glass pipette (20–30 µm tip diameter) with a micromanipulator (Narishige International USA Inc., Amityville, NY, USA) controlling the injection speed. Optical fibers were implanted bilaterally above LH^PV^ somas (bregma, −1.80 mm; midline, ± 1.40 mm; skull surface, −5.00 mm; no angle). For experiments targeting LH^PV^ axonal projections within the vlPAG, 40 nl of an adeno-associated virus (rAAV2/1-CAG-FLEX-*rev*-ChR2-tdTomato, titer: 6.9 × 10^12^ GC/ml or rAAV2/1-CAG-FLEX-tdTomato, titer: 4.5 × 10^12^ GC/ml) was injected bilaterally (rate: 25 nl/min) into the LH of *Pvalb*^*Cre*^ mice and optical fibers were implanted bilaterally at 10° angles above LH^PV^ axonal projections in the vlPAG (bregma, −3.87 mm; midline, ±0.84 mm; skull surface, −2.7 mm), previously described as the rostral portion of the PV1 terminal field within the Su3 region^16^. Fiber implants were affixed to the skull with cyanoacrylate adhesive and C&B Metabond Quick Adhesive Cement System (Parkell, Inc., Edgewood, NY, USA). Subsequently, mice were individually housed for three to four weeks for post-surgical recovery and viral transduction.

For brain slice electrophysiological recordings, two to five-month-old heterozygous *Pvalb*^*Cre*^ mice were bilaterally injected with 100 nl of an adeno-associated virus (rAAV2/1-CAG-FLEX-*rev*-ChR2:tdTomato, titer: 6.86 × 10^12^ GC/ml or rAAV2/9-CAG-FLEX-ArchT-GFP, titer: 4.7 × 10^12^ GC/ml) into the LH as described above. Recordings were performed 2–5 weeks after post-surgical recovery and viral transduction.

### Optical manipulations

Optical fiber implants were connected to patch cords which were connected to lasers (Doric Lenses Inc., Quebec, Canada) via rotary joints mounted over behavioral testing areas. Laser output was controlled by Doric Neuroscience Studio software (v5.1). For ChR2 and control experiments, 450 nm laser diodes were used to deliver 5-ms pulses of 10–15 mW blue light at a frequency of 50 Hz. For ArchT and control experiments, 520 nm laser diodes were used to deliver 10–15 mW of constant green light.

### Behavioral experiments

Mice were habituated to experimenter handling for 3 days prior to experiments. and all experiments were performed during the light cycle. Mice were acclimated to behavioral rooms for at least 1 h before experiments began. By design, each group contained 8 mice, and across experimental and control groups, mice were gender-matched and age-matched or littermates. Mice were excluded from analysis if viral expression and fiber placement were not observed in at least one hemisphere after histological assessment (see ***Histology***).

### Thermal nociception

A cylindrical plexiglass enclosure was placed on a 51 °C hot plate (IITC Life Science, Woodland Hills, CA, USA). Patch cords were connected, and mice were handled for an initial 3-min period. Mice were gently placed on the hot plate and the latency to paw withdrawal (PWL) was measured. Following this measurement, mice were removed from the hot plate and photostimulation commenced for 3 min during handling, after which mice were placed back on the hot plate for a second PWL measurement. For naltrexone experiments, 10 mg/kg naltrexone (CHEBI:134687; Sigma-Aldrich, St. Louis, MO, USA) was administered subcutaneously (s.c.) 30 min prior to the 6-min test.

To verify that 10 mg/kg naltrexone sufficiently antagonized µ-opioid receptors, a morphine cumulative dosing procedure was used in which PWL measurements were taken 30 min following each injection and each measurement was followed immediately by the next injection. First, saline was injected (s.c.), and in subsequent cycles, the following doses of morphine were administered (s.c.): 3.2 mg/kg, 6.8 mg/kg (for a cumulative dose of 10 mg/kg), and 22 mg/kg (for a cumulative dose of 32 mg/kg). Four days later, the same group of mice was retested as described above with the exception that the first injection was 10 mg/kg (s.c.) naltrexone instead of saline. Morphine was obtained from the National Institute on Drug Abuse Drug Supply Program (CHEBI:134687).

### Locomotion

Mice were connected to patch cords and placed into plastic open field chambers (30 × 27 × 30 cm) for 6 min, during which the second 3-min period was paired with photostimulation. The session was recorded and analyzed with ANY-maze v5 (RRID:SCR_014289; Stoelting Co., Wood Dale, IL, USA) software and the distance traveled during each 3-min period was calculated.

### Elevated plus maze (EPM)

A standard EPM apparatus consisting of two open arms (30 × 5 cm) and two closed arms (30 × 5 × 30 cm) extending from a central platform (5 × 5 cm) and elevated 75 cm from the floor was used. Mice were connected to patch cords and placed in the center of the EPM. The 6-min test session began immediately, during which the second 3-min period was paired with photostimulation. EthoVision XT (RRID:SCR_000441; Noldus, Wageningen, Netherlands) software was used to track and record the location of the mice during the test. Open arm time was calculated as the percentage of each 3-min period the mice spent in the open arms. Open arm and closed arm entries were defined as head entries from the central platform into the open arm or closed arm zones, respectively. Although we set an *a priori* exclusion criteria for mice that spent less than five percent time in the open arms during the first epoch, no mice required exclusion.

### Acetic acid-induced writhing

Mice were connected to patch cords before receiving intraperitoneal (i.p.) injections of 0.6% acetic acid (CHEBI:15366; Sigma-Aldrich, St. Louis, MO, USA), delivered in a volume of 10 ml/kg. Mice were then placed into a plexiglass observation cage similar in dimension to their home cages and recorded for 30 min, during which photostimulation occurred for alternating 3-min periods. Whether the first period was laser ON or OFF was randomized and counterbalanced across groups. Writhing behavior, defined by body contortions and extension of the hind limbs, was subsequently scored in 3-min bins by an observer blinded to the experimental conditions.

### Histology

Mice were deeply anesthetized with isoflurane and transcardially perfused with 1x phosphate buffered saline (PBS) followed by 4% paraformaldehyde (PFA) in 1x PBS. Whole brains were removed and post-fixed in 4% PFA overnight at 4 °C and subsequently transferred to 1x PBS for storage at 4 °C until further processing. Coronal brain sections (50 µm thick) were collected in 1x PBS using a Leica VT1200 vibratome (Leica Biosystems GmBH, Wetzlar, Germany). Sections were mounted with DAPI-Fluoromount-G aqueous mounting medium (Electron Microscopy Sciences, Hatfield, PA, USA) onto Superfrost Plus glass slides (VWR International, Radnor, PA, USA). Images were taken with an AxioZoom.V16 fluorescence microscope (Carl Zeiss Microscopy LLC, Thornwood, NY, USA).

### Patch clamp electrophysiology

For channelrhodopsin (ChR2)-assisted circuit mapping (CRACM) of LH^PV^ neurons synaptically connected to vlPAG neurons, mice were bilaterally injected with an adeno-associated virus (AAV2/1) into the LH (see Stereotaxic Viral Injection). Neurons were visualized with infrared differential interference contrast (IR-DIC) optics on an AxioExaminer.Z1 microscope (Carl Zeiss Microscopy LLC). Whole-cell voltage-clamp recordings of vlPAG neurons were performed using patch pipettes (3.3–4.0 MΩ) containing (in mM): 117 cesium methanesulfonate, 20 HEPES, 0.4 EGTA, 2.8 NaCl, 5 TEA-Cl, 4 Mg-ATP, 0.4 Na-GTP, and 0.2% biocytin (pH adjusted to 7.3 using CsOH, and osmolality of 281 mOsm), while current-clamp recordings of LH^PV^ neurons were performed using (in mM) 135 potassium gluconate, 10 HEPES, 4 KCl, 4 MgATP, 0.3 Na3GTP, and 0.2% biocytin (pH adjusted to 7.3 using KOH, and osmolality of 290 mOsm/kg H_2_O). Recordings were performed using a Multiclamp 700B amplifier (1 kHz low-pass Bessel filter and digitized at 10 kHz using a Digidata 1440A). Photocurrents were evoked by 5 ms blue (470 nm) light pulses (light emitting diode M470L3; Thorlabs, Inc., Newton, NJ, USA). Light-evoked excitatory currents (EPSCs) were blocked by perfusing 10 μM DNQX and 50 μM APV (AMPA-R and NMDA-R antagonists). All recordings were made at 32 °C. All chemicals were obtained from Sigma-Aldrich (MO, USA) or Tocris Bioscience (Bristol, UK).

For archaerhodopsin (ArchT) recordings, mice were bilaterally injected with an adeno-associated virus (AAV2/9) into the LH of *Pvalb*^*Cre*^ mice (see Stereotaxic Viral Injection) and whole-cell current-clamp and voltage-clamp recordings of LH^PV^ neurons were performed as described above with the following changes. Photocurrents were evoked by 20 ms or 500 ms yellow (590 nm) light pulses (light emitting diode M590L3; Thorlabs, Inc.).

### Quantification and statistical analysis

Graphs and statistics for behavioral experiments were prepared with GraphPad Prism 8 software (RRID:SCR_002798; GraphPad, La Jolla, CA, USA). All data are plotted as mean ± s.e.m. F tests did not detect differences in variance for any results shown. Additionally, residual plots and QQ plots appeared normal for all data. Finally, Shapiro-Wilk tests confirmed normal distribution of all residuals. Optogenetic behavioral data from thermal nociception, locomotion, and elevated plus maze experiments were first analyzed by subtracting the measurement during the first 3-min laser OFF epoch from the second 3-min laser ON epoch (*e.g*. ∆ paw withdrawal latency = paw withdrawal latency during photostimulation – paw withdrawal latency before photostimulation). Two-tailed, unpaired Student’s *t* tests or one-way ANOVA with Bonferroni’s multiple comparisons test were then used for between-group comparisons. For the experiment with morphine and naltrexone, absolute paw withdrawal latency values were analyzed with two-way repeated measures ANOVA with Bonferroni’s multiple comparisons test. For the acetic acid-induced writhing experiment, the number of writhes observed during laser OFF epochs was subtracted from writhes observed during the ON epochs, and a two-tailed, unpaired Student’s *t* test was used to compare between groups. Data from mice were excluded from analysis if viral expression and fiber placement were mistargeted or were not observed in at least one hemisphere after histological assessment (excluded mice: *n* = 2 LH^PV^:ChR2, *n* = 1 LH^PV^:ArchT, *n* = 2 LH^PV^:ChR2→vlPAG). Additionally, accurate hot plate measurements could not be determined for one of the remaining LH^PV^:ChR2→vlPAG mice, leading to a group size of 5 in the hot plate test but otherwise a group size of 6 in the other assays for the LH^PV^:ChR2→vlPAG group. Data from electrophysiological recordings were analyzed with Clampfit v10.6 (RRID:SCR_011323; Molecular Devices LLC, San Jose, CA, USA) and Origin Pro v9.2 (RRID:SCR_014212; OriginLab Corporation, Northampton, MA, USA). Peak current amplitude was measured with Clampfit v10.6 using the average of ten photostimulation sweeps. For all experiments, *p* < 0.05 was considered significant.

## Supplementary information

Supplementary Figures S1 - S3

## Data Availability

All data supporting the findings of this study are available from the corresponding author upon reasonable request.
