# Supplementary material for: Lateral hypothalamic fast-spiking parvalbumin neurons modulate nociception through connections in the periaqueductal gray area
Source: Sci Rep. 2019 Aug 19;9:12026. doi: 10.1038/s41598-019-48537-y (PMC6700312; doi:10.1038/s41598-019-48537-y)
Supplement: Supplementary file 1 — Supplementary Figures S1 - S3 [file 41598_2019_48537_MOESM1_ESM.pdf]

Supplementary Information

Lateral hypothalamic fast-spiking parvalbumin neurons modulate nociception through connections in the periaqueductal gray area

Justin N. Siemian[1], Cara B. Borja[1], Sarah Sarsfield[1], Alexandre Kisner[1] & Yeka Aponte[1,2]

[1]Neuronal Circuits and Behavior Unit, National Institute on Drug Abuse Intramural Research Program, National Institutes of Health, Baltimore, MD 21224-6823, USA

[2]The Solomon H. Snyder Department of Neuroscience, Johns Hopkins University School of Medicine, Baltimore, MD 21205, USA

Supplementary Figure S1

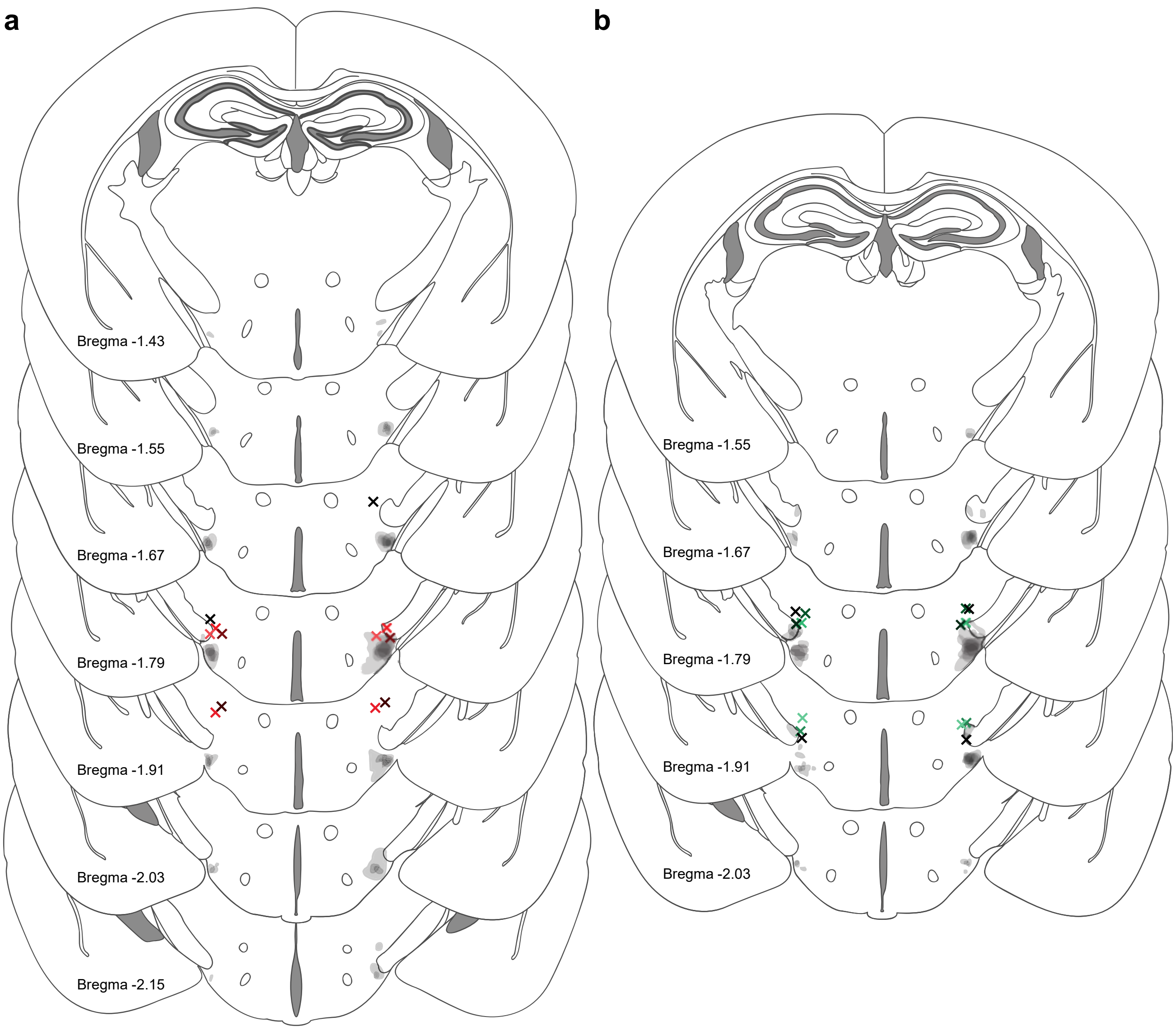

(**a-b**) Schematic representations of (**a**) AAV-FLEX-ChR2:tdTomato and (**b**) AAV-FLEX-ArchT:GFP viral expression in the LH of *Pvalb*^{Cre} transgenic mice. Optical fibers (×) were implanted in the LH above viral targets. Schematic images adapted from Franklin and Paxinos (Fourth edition, 2013).

Supplementary Figure S2

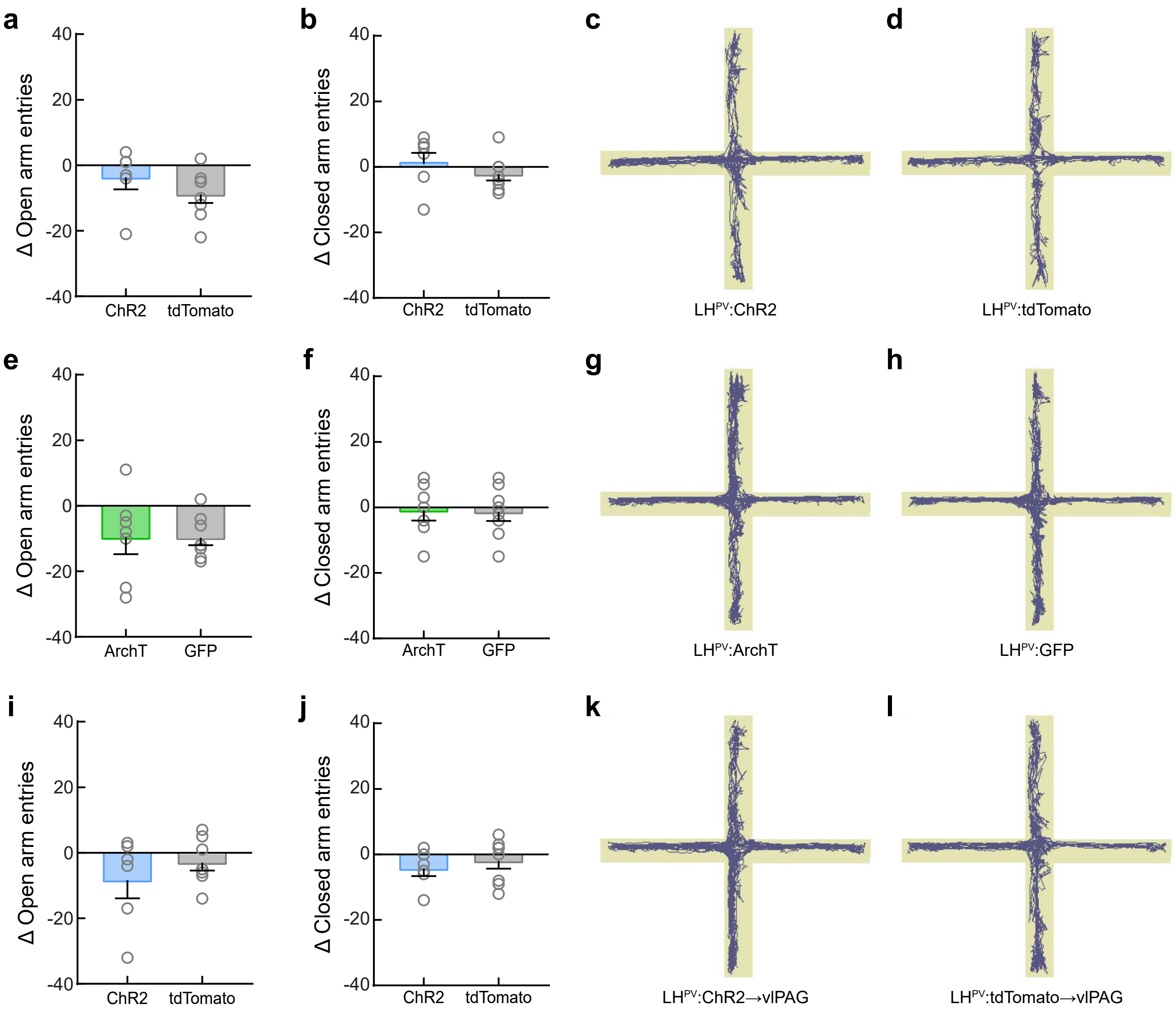

Optogenetic LH^{PV} neuronal manipulations do not affect elevated plus maze open or closed arm entries. (**a**) Photostimulation of LH^{PV}:ChR2 mice and LH^{PV}:tdTomato control mice did not alter open arm entries (unpaired Student's *t* test, $t(12) = 1.18$, $p = 0.26$) or (**b**) closed arm entries ($t(12) = 1.07$, $p = 0.30$). (**c-d**) Representative EPM traces for LH^{PV}:ChR2 and LH^{PV}:tdTomato mice. (**e**) Photoinhibition of LH^{PV}:ArchT mice and LH^{PV}:GFP control mice did not alter open arm entries ($t(13) = 0.007$, $p = 0.99$) or (**f**) closed arm entries ($t(13) = 0.12$, $p = 0.90$). (**g-h**) Representative EPM traces for LH^{PV}:ArchT and LH^{PV}:GFP mice. (**i**) Photostimulation of LH^{PV}:ChR2→vlPAG mice and LH^{PV}:tdTomato→vlPAG control mice did not alter open arm entries ($t(12) = 0.96$, $p = 0.35$) or (**j**) closed arm entries ($t(12) = 0.69$, $p = 0.50$). (**k-l**) Representative EPM traces for LH^{PV}:ChR2→vlPAG mice and LH^{PV}:tdTomato→ vlPAG mice.

Supplementary Figure S3

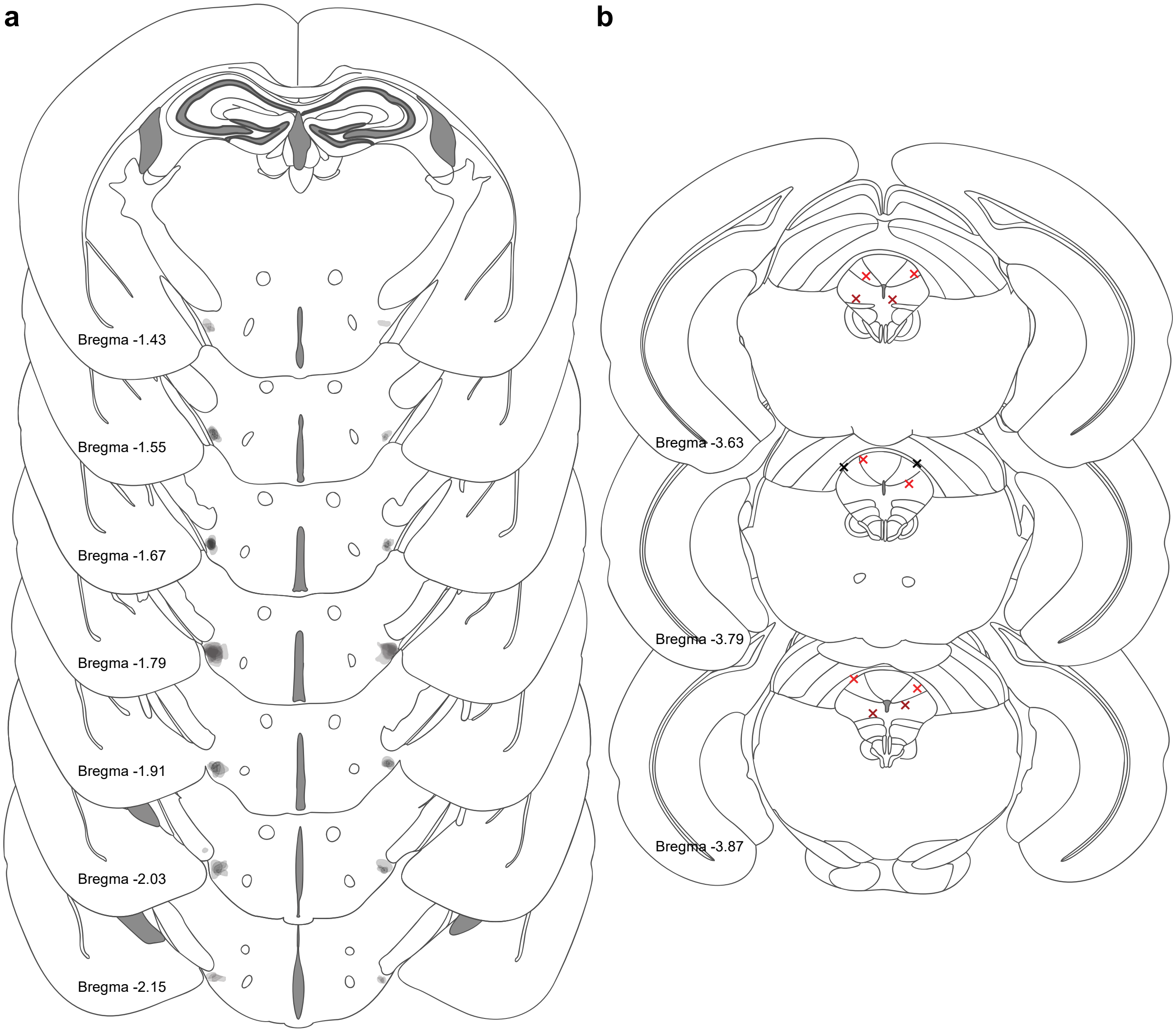

(**a-b**) Schematic representations of (**a**) AAV-FLEX-ChR2:tdTomato viral expression in the LH of *Pvalb*^{Cre} transgenic mice and (**b**) optical fiber (×) placement above the vlPAG. Schematic images adapted from Franklin and Paxinos (Fourth edition, 2013).
